# Establishment of a Prediction Model Based on Preoperative MRI Radiomics for Diffuse Astrocytic Glioma, IDH-Wildtype, with Molecular Features of Glioblastoma

**DOI:** 10.3390/cancers15205094

**Published:** 2023-10-21

**Authors:** Peng Du, Xuefan Wu, Xiao Liu, Jiawei Chen, Aihong Cao, Daoying Geng

**Affiliations:** 1 Department of Radiology, Huashan Hospital, Fudan University, Shanghai 200040, China; 2 Department of Radiology, The Second Affiliated Hospital of Xuzhou Medical University, Xuzhou 221000, China; 3 Shanghai Gamma Hospital, Shanghai 200040, China; guoy19@fudan.edu.cn; 4 School of Computer and Information Technology, Beijing Jiaotong University, Beijing 100044, China; 5 Department of Neurosurgery, Huashan Hospital, Shanghai 200040, China; 20111220062@fudan.edu.cn

**Keywords:** MRI, radiomics, diffuse astrocytic glioma, IDH-wildtype, glioblastoma, DAG-G, TERT promoter mutation, EGFR amplification

## Abstract

**Simple Summary:**

The prediction model established based on preoperative MRI radiomics in this study can basically realize the prospective, non-invasive, and accurate diagnosis of diffuse astrocytic glioma, IDH wild-type, with molecular features of glioblastoma, WHO grade 4 (DAG-G), which is of great significance to help optimize the treatment plan for such patients, including expanding the extent of surgery and actively administering radiotherapy, targeted therapy, or other treatments after surgery, so as to fundamentally maximize the prognosis of patients.

**Abstract:**

Purpose: In 2021, the WHO central nervous system (CNS) tumor classification criteria added the diagnosis of diffuse astrocytic glioma, IDH wild-type, with molecular features of glioblastoma, WHO grade 4 (DAG-G). DAG-G may exhibit the aggressiveness and malignancy of glioblastoma (GBM) despite the lower histological grade, and thus a precise preoperative diagnosis can help neurosurgeons develop more refined individualized treatment plans. This study aimed to establish a predictive model for the non-invasive identification of DAG-G based on preoperative MRI radiomics. Patients and Methods: Patients with pathologically confirmed glioma in Huashan Hospital, Fudan University, between September 2019 and July 2021 were retrospectively analyzed. Furthermore, two external validation datasets from Wuhan Union Hospital and Xuzhou Cancer Hospital were also utilized to verify the reliability and accuracy of the prediction model. Two regions of interest (ROI) were delineated on the preoperative MRI images of the patients using the semi-automatic tool ITK-SNAP (version 4.0.0), which were named the maximum anomaly region (ROI1) and the tumor region (ROI2), and Pyradiomics 3.0 was applied for feature extraction. Feature selection was performed using a least absolute shrinkage and selection operator (LASSO) filter and a Spearman correlation coefficient. Six classifiers, including Gauss naive Bayes (GNB), K-nearest neighbors (KNN), Random forest (RF), Adaptive boosting (AB), and Support vector machine (SVM) with linear kernel and multilayer perceptron (MLP), were used to build the prediction models, and the prediction performance of the six classifiers was evaluated by fivefold cross-validation. Moreover, the performance of prediction models was evaluated using area under the curve (AUC), precision (PRE), and other metrics. Results: According to the inclusion and exclusion criteria, 172 patients with grade 2–3 astrocytoma were finally included in the study, and a total of 44 patients met the diagnosis of DAG-G. In the prediction task of DAG-G, the average AUC of GNB classifier was 0.74 ± 0.07, that of KNN classifier was 0.89 ± 0.04, that of RF classifier was 0.96 ± 0.03, that of AB classifier was 0.97 ± 0.02, that of SVM classifier was 0.88 ± 0.05, and that of MLP classifier was 0.91 ± 0.03, among which, AB classifier achieved the best prediction performance. In addition, the AB classifier achieved AUCs of 0.91 and 0.89 in two external validation datasets obtained from Wuhan Union Hospital and Xuzhou Cancer Hospital, respectively. Conclusions: The prediction model constructed based on preoperative MRI radiomics established in this study can basically realize the prospective, non-invasive, and accurate diagnosis of DAG-G, which is of great significance to help further optimize treatment plans for such patients, including expanding the extent of surgery and actively administering radiotherapy, targeted therapy, or other treatments after surgery, to fundamentally maximize the prognosis of patients.

## 1. Introduction

With the rapid development of pathology and molecular detection technologies, an increasing amount of genetic and molecular information has been detected, identified, and applied in clinical practice to guide the diagnosis and treatment of diseases. The 2021 World Health Organization (WHO) classification of central nervous system (CNS) tumors has developed a new tumor classification system by integrating the latest research progress and seven updates of the consortium to Inform Molecular and Practical Approaches to CNS Tumor Taxonomy (cIMPACT-NOW), with a special emphasis on promoting molecular diagnosis of CNS tumors, and put forward the concept of the integrated diagnosis of brain tumors [1], which is in line with the requirements of precision medicine and individualized medicine.

In the updated classification criteria [2], IDH wild-type grade 2–3 diffuse astrocytic glioma without necrosis or microvascular proliferation is also diagnosed as glioblastoma (GBM), IDH wild-type, or WHO grade 4, if any one or combinations of the three conditions of TERT promoter mutation, EGFR amplification, and chromosome 7 amplification/chromosome 10 deletion are met. The treatment principles recommended are the same as those for GBM, which are also known as diffuse astrocytic glioma, IDH wild-type, with molecular features of glioblastoma, or WHO grade 4 (DAG-G). It has been shown [3,4,5] that DAG-G is an early stage of histological GBM, which will eventually develop into the typical GBM histological features.

Therefore, for IDH wild-type WHO grade 2–3 diffuse astrocytic glioma with low histological grade and consistent molecular characteristics of GBM, it is of extreme significance to obtain non-invasive and precise identification preoperatively. This can not only help neuro-oncologists to formulate more refined individualized treatment plans, including expanding the scope of surgical resection, and actively performing postoperative radiotherapy, targeted therapy, or other treatments after surgery, which can maximally improve the prognosis of patients, but also enable patients and their families to further understand the disease and have certain knowledge about the possible poor prognosis, thereby avoiding possible misunderstandings and contradictions to a certain extent. However, there are few studies on the use of radiomics in the preoperative prediction of DAG-G.

In this study, a molecular staging prediction model for low-grade diffuse astrocytic gliomas was established based on preoperative multi-modal MRI radiomics to predict IDH wild-type, TERT promoter mutation, and EGFR amplification status; finally, the prediction results were integrated for the diagnosis of DAG-G.

## 2. Patients and Methods

### 2.1. Patients

This study was approved by the Institutional Review Board of Huashan Hospital, Fudan University (Number: KY2021-066). Data of pathologically confirmed glioma patients from Huashan Hospital, Fudan University between September 2019 and July 2021 were retrospectively analyzed. Furthermore, two datasets of glioma patients from Wuhan Union Hospital and Xuzhou Cancer Hospital, respectively, were also applied for external validation.

### 2.2. Inclusion and Exclusion Criteria

Inclusion criteria: (1) patients aged over 18 years; (2) patients with histopathological diagnosis of grade 2–3 diffuse astrocytic gliomas; (3) patients with complete postoperative molecular genetics information (IDH, TERT promoter mutation, and EGFR amplification); (4) patients who received brain MRI examination one week before surgery; (5) patients receiving brain MRI with a 3.0T scanner (Discovery MR750W; GE Healthcare, Milwaukee, WI, USA), with the sequences including T1 weighted imaging (T1WI), T2 weighted imaging (T2WI), T2-fluid attenuated inversion recovery (T2-Flair), diffusion-weighted imaging (DWI), and CE-T1WI.

Exclusion criteria: (1) previous history of brain tumors; (2) lesion was predominant hemorrhage; (3) artifacts on MRI images; (4) other treatment before surgery; (5) incomplete clinical data.

## 3. MRI Scanning Parameters

Brain MRI scanning parameters are shown in Table 1. The scanning range of all sequences covered the entire brain. The contrast agent, Gadodiamide injection (GE Pharmaceuticals, Milwaukee, WI, USA), was injected through the elbow vein at a dose of 0.1 mmol per kilogram of body weight for CE-T1WI scanning. After the injection of the contrast agent, a transverse CE-T1WI scanning was initiated immediately, and after the injection of the contrast agent, and 20 mL physiological saline was used for rinsing.

## 4. Pathological Grading and Molecular Diagnosis

In this study, the histological grading and molecular genetic diagnosis of glioma were completed by the Department of Pathology of Huashan Hospital. Moreover, IDH1/IDH2 gene mutation/wild-type and TERT promoter mutation were detected by Sanger sequencing, and EGFR amplification was detected through fluorescence in situ hybridization (FISH).

## 5. Data Preprocessing

First, an anonymous operation was carried out on the image information of enrolled patients. Afterward, to match the region of interest (ROI) with the images of all sequences, the linear differential resampling means in SimpleITk software package (version 2.1.1.1, https://simpleitk.readthedocs.io/en/master/index.html (accessed on 5 August 2023)) was used to resample all images to 240 × 240 × 24 with an interval of 1 × 1 × 4 mm^3^. Thereafter, by utilizing ANTs (https://github.com/ANTsX (accessed on 5 August 2023)), all sequences (T1WI, T2WI, T2-Flair, and DWI) were registered to CE-T1WI. Subsequently, SimpleITK was adopted to normalize the image grayscale value to 0–255.

## 6. Image Segmentation

**Figure 1:**
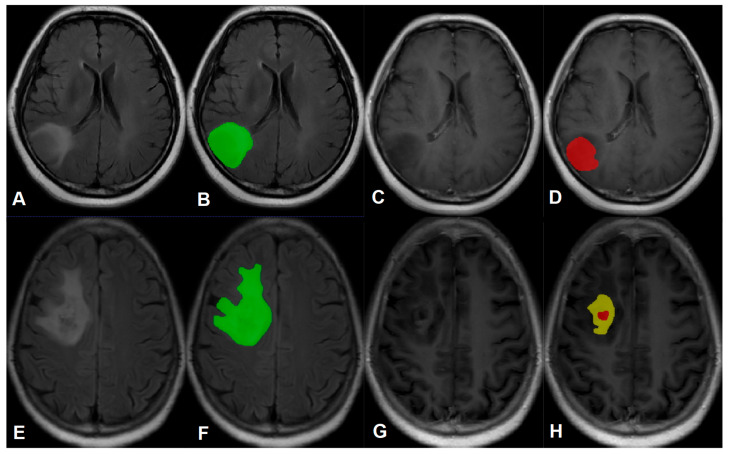
(**A**,**B**,**E**,**F**) are T2-Flair images, and (**C**,**D**,**G**,**H**) are CE-T1WI images. (**A**–**D**) A case of WHO grade 2 astrocytoma, B represents the maximum anomaly region (ROI1) delineated in green; D represents the tumor region (ROI2), red represents the non-enhancement region. (**E**–**H**) A case of WHO grade 3 astrocytoma, F represents the maximum anomaly region (ROI1) delineated in green; H represents the tumor region (ROI2), yellow represents the enhancement region, and red represents the non-enhancement region.

The MRI image segmentation of tumors was implemented based on the research of Zacharaki et al. [6], and was performed by a radiologist with more than 15 years of experience in medical imaging diagnosis. To be specific, the tumors were segmented on the T2-Flair and CE-T1WI axial images by utilizing ITK-SNAP (version 4.0.0, http://www.itksnap.org/pmwiki/pmwiki.php (accessed on 5 August 2023)). Afterward, all tumors were delineated in two parts, namely, ROI1 and ROI2: (1) ROI1 represented the maximum anomaly region (MAR), and was delineated on the T2-Flair image, represented in green; (2) ROI2 represented the tumor area (Tumor), and was delineated on CE-T1WI image, with yellow representing the enhancement area and red representing the non-enhancement area. A representative tumor ROI delineation is displayed in Figure 1.

## 7. Feature Extraction and Selection

A total of 12,918 features were extracted using Pyradiomics (version 3.0, https://pyradiomics.readthedocs.io/en/latest/features.html (accessed on 5 August 2023)) for 6 combinations of 2 ROIs and 3 sequences, so as to force the extraction of 2D features with slice-averaged features instead of 3D features. Firstly, the most important features were selected utilizing the LASSO filter, and then the redundant features were removed by using the Spearman correlation coefficient method to remove features with high linear correlation.

## 8. Classifier Evaluation and Statistical Analysis

We used Python (version 3.8.0, https://www.python.org/ (accessed on 5 August 2023)) and Scikit-learn (version 1.0.2, https://scikit-learn.org/1.0/index.html (accessed on 5 August 2023)). The six classifiers were directly defined, called, trained, and saved by Scikit-learn:

GNB = GaussianNB ()
KNN = KneighborsClassifier ()
RF = RandomForestClassifier (n_estimators = 100, criterion = ‘entropy’, max_depth = 5)
AB = AdaBoostClassifier (DecisionTreeClassifier (max_depth = 3), n_estimators = 100,learning_rate = 1, algorithm = “SAMME”)
SVM = SVC (kernel = ‘linear’, C = 1, decision_function_shape = ‘ovr’, probability = True)
MLP = MLPClassifier (random_state = 1, max_iter = 500)

Prediction models were constructed using the following six classifiers, including Gauss naive Bayes (GNB), K-nearest neighbors (KNN), Random forest (RF), Adaptive boosting (AB), and Support vector machine (SVM) with linear kernel and multilayer perceptron (MLP). The prediction performances of these six classifiers were evaluated by a 5-fold cross-validation method. Meanwhile, the area under the receiver operating characteristic (ROC) curve (AUC), precision (PRE), recall (REC), and F1-score were utilized to evaluate the performance of each prediction model.

## 9. Results

### Baseline Characteristics

According to the inclusion criteria, 193 patients with astrocytic glioma were recruited in this study, among them, 21 cases were excluded due to a previous history of intracranial tumor (*n* = 3), the presence of predominant hemorrhage lesions (*n* = 5), the presence of artifacts on MRI images (*n* = 7), preoperative treatment (*n* = 2), and incomplete clinical data (*n* = 4). Ultimately, 172 patients were included in the study, with a median age of 47.97 ± 13.81 years. There were 103 males and 69 females, including 105 of grade 2 and 67 of grade 3. Meanwhile, there were 85 IDH-mutant cases and 87 IDH wild-type cases, 66 of them carried TERT promoter mutation, whereas 17 carried EGFR amplification. The baseline characteristics of enrolled patients are presented in Table 2.

**Figure 2:**
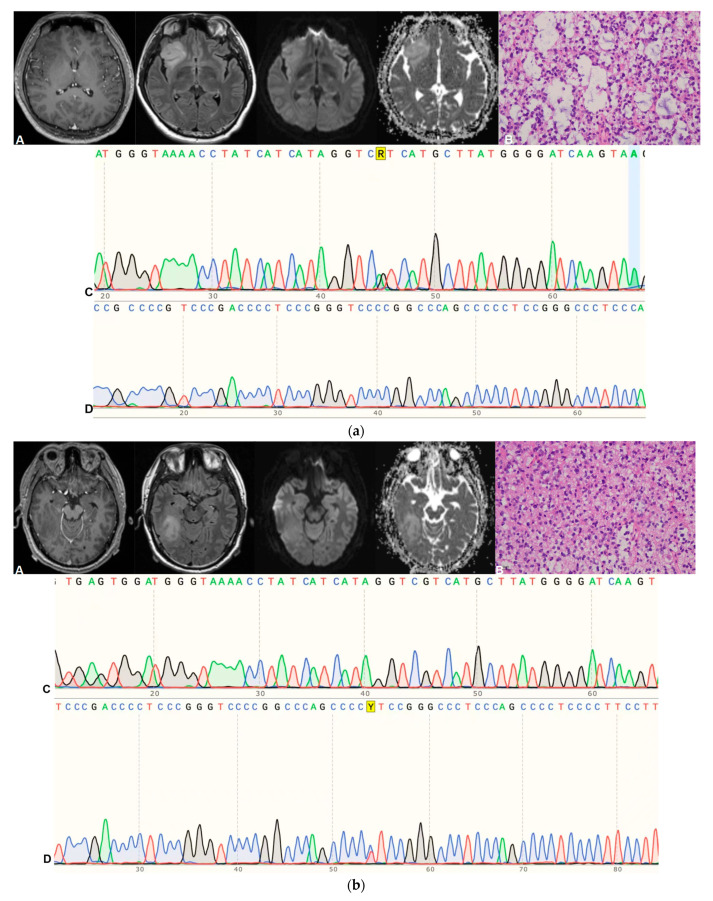

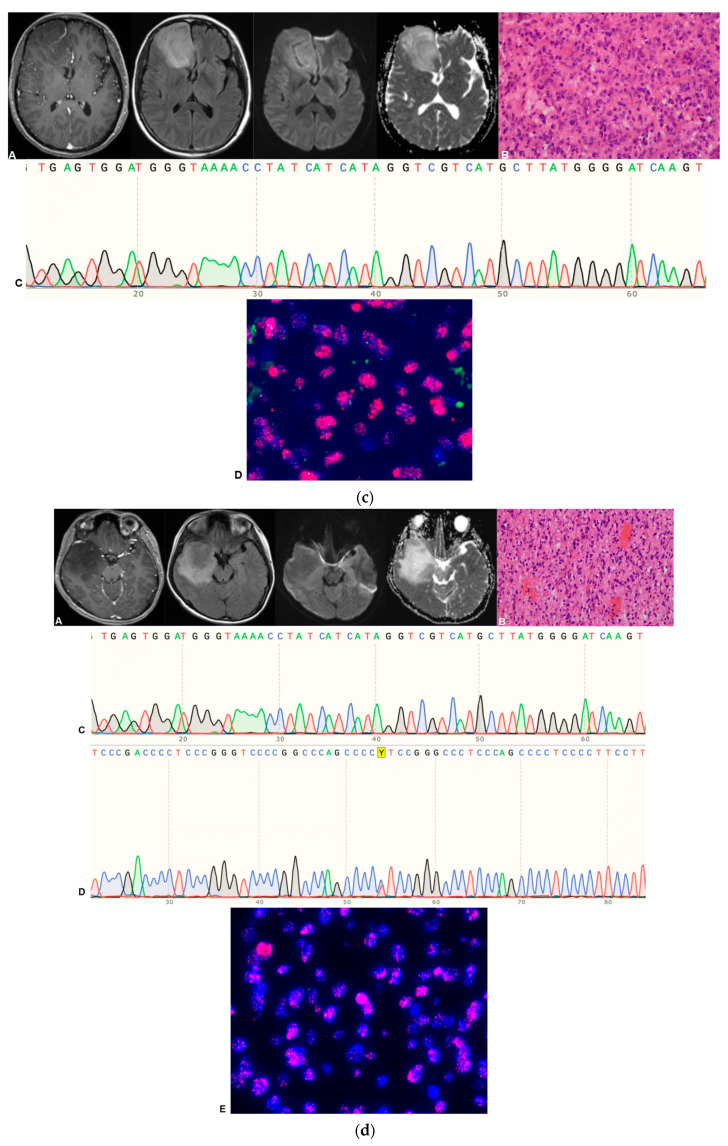
(**a**) A case of WHO grade 2 astrocytoma with IDH-mutant and TERT promoter wild-type. A are MRI images (From left to right: CE-T1WI, T2-Flair, DWI, and ADC); B is pathology HE stain; C is IDH R132H mutant; D is TERT promoter wild-type. (**b**) A case of WHO grade 2 astrocytoma with IDH-wildtype and TERT promoter mutant. A are MRI images (From left to right: CE-T1WI, T2-Flair, DWI, and ADC); B is pathology HE stain; C is IDH1 wild-type; D is TERT C228T mutant. (**c**) A case of WHO grade 3 astrocytoma with IDH-wildtype and EGFR amplification. A are MRI images (From left to right: CE-T1WI, T2-Flair, DWI, and ADC); B is pathology HE stain; C is IDH1 wild-type; D is EGFR amplification. (**d**) A case of WHO grade 3 astrocytoma with IDH-wildtype, TERT promoter mutant, and EGFR amplification. A are MRI images (From left to right: CE-T1WI, T2-Flair, DWI, and ADC); B is pathology HE stain; C is IDH1 wild-type; D is TERT C228T mutant; E is EGFR amplification.

In total, 44 patients, including 30 males and 14 females, met the diagnosis of DAG-G, with a mean age of 46.58 ± 12.55 years. Of them, 20 cases were of grade 2 and 24 of grade 3; in addition, there were 31 cases with TERT promoter mutation, 14 with EGFR amplification, and only one with both TERT promoter mutation and EGFR amplification. Some typical cases of DAG-G are shown in Figure 2a–d.

In addition, based on the inclusion and exclusion criteria, an additional 32 glioma patients from Wuhan Union Hospital and 21 from Xuzhou Cancer Hospital were enrolled as the external validation datasets, and 9 and 5 patients met the diagnosis of DAG-G, respectively.

## 10. Feature Selection

**Figure 3:**
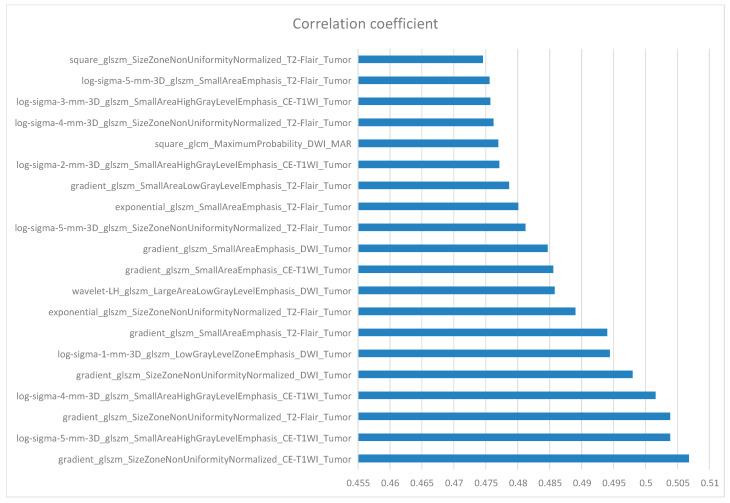
The 20 most relevant radiomics features for the IDH mutation prediction task.

**Figure 4:**
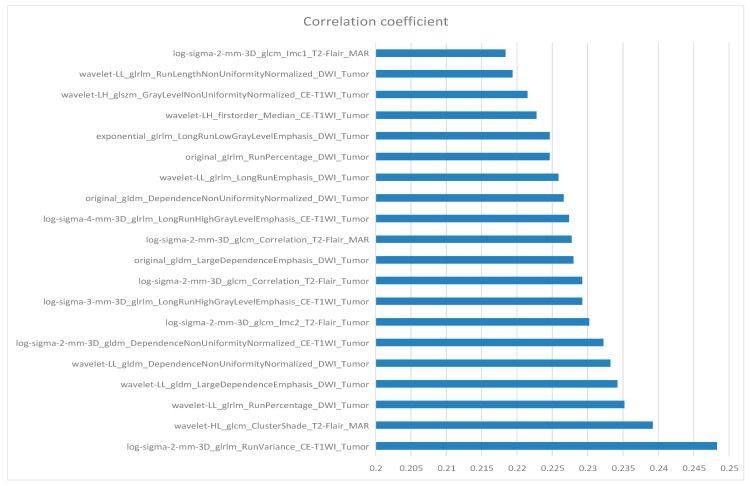
The 20 most relevant radiomics features for the TERT promoter mutation prediction task.

**Figure 5:**
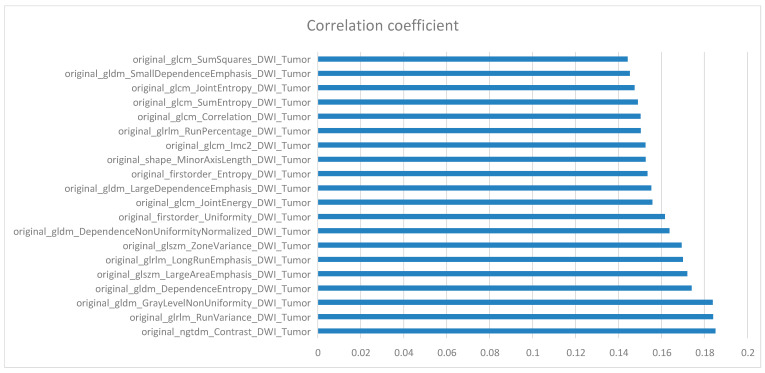
The 20 most relevant radiomics features for the EGFR amplification prediction task.

Through the IDH mutation prediction task, 165 features were screened, and the top 20 features with the strongest correlation are shown in Figure 3. According to the regional distribution, most of these features were from the tumor region. According to the sequence distribution, there were 6, 9, and 5 features extracted from CE-T1WI, T2-Flair, and DWI sequences, respectively. Moreover, 57 features were screened from the TERT promoter mutation prediction task, and the top 20 features with the strongest relevance are presented in Figure 4. According to the regional distribution, 17 features were from the tumor region, while 3 were from the maximum anomaly region, and there were 6, 5, and 9 features from the CE-T1WI, T2-Flair, and DWI sequences, respectively, according to the sequence distribution. From the EGFR amplification prediction task, 78 features were screened, and the top 20 most relevant features were all derived from tumor region and DWI sequence, as shown in Figure 5.

## 11. Performance of the Prediction Models

**Figure 6:**
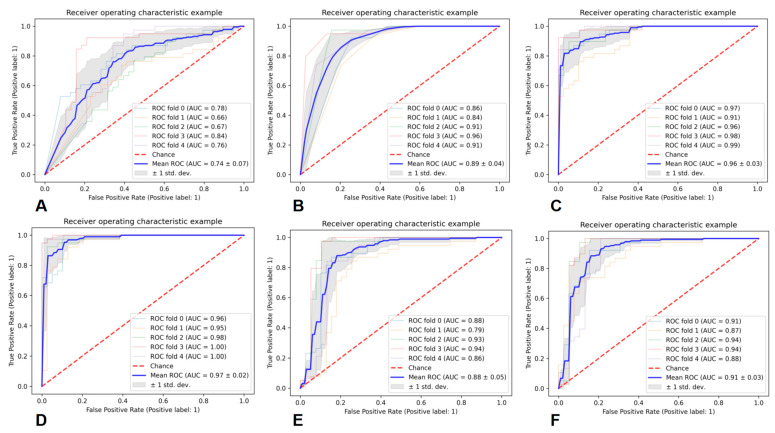
ROC curves of six classifiers for predicting DAG-G, (**A**) is GNB, (**B**) is KNN, (**C**) is RF, (**D**) is AB, (**E**) is SVM, and (**F**) is MLP.

Six classifiers were used for the prediction of DAG-G. As a result, the average AUC of GNB was 0.74 ± 0.07, that of KNN was 0.89 ± 0.04, that of RF was 0.96 ± 0.03, that of AB was 0.97 ± 0.02, that of SVM was 0.88 ± 0.05, and that of MLP was 0.91 ± 0.03. The above results showed that the AB classifier achieved the best performance in predicting DAG-G. The specific evaluation parameters are presented in Table 3, and the ROC curves of the six classifiers are shown in Figure 6.

In external validation, the AB classifier achieved an AUC of 0.91, a PRE of 0.86, a REC of 0.83, and a F_1_-score of 0.84 based on the dataset from Wuhan Union Hospital; while it achieved an AUC of 0.89, a PRE of 0.87, a REC of 0.75, and a F_1_-score of 0.81 based on the dataset from Xuzhou Cancer Hospital.

## 12. Discussion

Studies have suggested that the molecular genetic basis of glioma has a critical impact on the definition of tumor type and patient prognosis [7,8,9,10], and that genetic markers reflecting glioma type may be more closely related to overall survival than the previously used histopathological classification [11,12]. Since 2016, WHO started to decipher the spectrum and classification of gliomas using key molecular markers such as IDH mutation [13]. However, subsequent studies have discovered that gliomas classified based on IDH wild-type and IDH-mutant show further prognostic differences in patients, since they may contain other molecular genetic variants [14]. There is evidence supporting that although the histopathological status is “benign”, grade 2–3 diffuse astrocytic gliomas with GBM molecular characteristics exhibit similar manifestations to GBM as defined by classical histopathology [12]. In 2018, cIMPACT-NOW published its third update establishing a new diagnosis of diffuse astrocytic glioma, IDH wild-type, with GBM molecular features, WHO grade 4 [11]. This diagnosis focuses on the clinical prognostic differences observed in histological grade 2–3 IDH wild-type diffuse astrocytic gliomas, and gliomas meeting this diagnosis need to contain at least one of the following criteria: (1) TERT promoter mutation, (2) chromosome 7 amplification/chromosome 10 deletion, and (3) EGFR amplification. As reported in studies [15], low-grade gliomas with such molecular features exhibit a clinical course similar to that of GBM. As a result, the fifth edition of the WHO CNS tumor classification criteria in 2021 introduced the concept of DAG-G.

In practice, neuro-oncologists and radiologists are increasingly encountered with a problem of poor prognosis of low histological grade gliomas, and the introduction of DAG-G may answer some of these questions. Referring to the statistics of the Chinese Glioma Genome Atlas from 2011 to 2017 [16], the mean age of onset of low-grade glioma (IDH wild-type) was 38.55 years, while that of GBM (IDH wild-type) was 52.86 years. In this study, the mean age of onset of DAG-G was 46.58 years, which was between the above two. Furthermore, it has been suggested that the mean age of onset of DAG-G is 64–67 years, older than that of IDH mutant astrocytic gliomas [17]. Studies have reported that the risk of epilepsy in gliomas is closely related to the primary location of the tumor [18,19,20]. In our cohort, more than half of the tumors were present in these epileptogenic-sensitive areas. We found that epilepsy was the most common clinical manifestation in patients with DAG-G in this study, followed by headache. In this study, 57% of patients experienced epilepsy, similar to the research results of Tesileanu et al. [21], and 16% of them experienced headaches, consistent with the research results of Lee et al. [14] and Grogan et al. [17]. We believe that the current clinical research on DAG-G is still in the initial stage, and there is a defect in the small sample size of the enrolled patients. Therefore, the analysis results may not be representative and instructive. In the future, larger sample sizes and patient data information from multiple centers are needed.

As discovered in studies, the radiological and histopathological features of DAG-G can hardly be distinguished from traditional IDH mutant astrocytic gliomas. The radiological features include a lack of calcification, mild space-occupying effects, small peri-tumoral edema, and no/mild enhancement. Meanwhile, the histopathological features include a lack of microvascular proliferation and palisade necrosis, as well as a large number of swollen astrocytomas [22]. Therefore, molecular testing and genetic analysis of gliomas are essential to detect and diagnose DAG-G regardless of the clinical presentations, radiological signs, or histopathological findings. However, for the time being, molecular testing and genetic analysis must be based on the invasive acquisition of tumor specimens by surgery/puncture and cannot be determined preliminarily. This has prevented the development of more individualized and refined treatment plans for patients who may require more aggressive or even radical treatment than other low-grade gliomas from the outset, for the sake of delaying recurrence and improving prognosis [17,23].

Given the limited radiological and microscopic signs of gliomas that can be identified by the human eyes, it is necessary to more accurately identify molecular information with other more advanced and objective methods and tools. Considering the ability to reveal textural and other higher-order features in medical images, radiomics may play a greater role in the diagnosis and identification of DAG-G. Currently, only Park et al. [24] conducted a relevant study in which they used a machine learning classifier based on preoperative MRI Visual AcceSAble Rembrandt Images (VASARI) and radiomics features to identify IDH wild-type low-grade gliomas with GBM molecular features, and obtained satisfactory classification results in the test set (AUC: 0.854). Nonetheless, the patients included in their study had both astrocytomas and oligodendrogliomas, but the WHO classification criteria for the diagnosis of DAG-G referred specifically to IDH wild-type grade 2–3 astrocytomas. In addition, compared with their study, VASARI features were not used in our study, instead, we added CE-T1WI sequence features and feature extraction of different regions of the tumor. In our study, the AB classifier attained the best prediction performance for DAG-G, with an average AUC of 0.97 ± 0.02.

The prediction model established based on preoperative MRI radiomics in this study basically realizes the prospective, non-invasive, and accurate preoperative diagnosis of DAG-G, which assists patients and doctors in winning the initiative of treatment to a certain extent, and helps doctors to formulate more detailed individualized treatment plans, including expanding the extent of surgery, and actively administering aggressive postoperative radiotherapy, targeted therapy or other treatments. Moreover, it also helps patients and their family members to have a better understanding of the disease and a certain knowledge of the possible poor prognosis, thereby avoiding the possible misunderstandings and contradictions in the follow-up, and is of greater clinical significance.

However, some inevitable limitations should be noted in this study. Firstly, this study was a single-center retrospective study with a small sample size, which inevitably leads to bias. Therefore, larger, prospective, and multi-centered studies are needed in the future to further verify the prediction performance of the model. Secondly, chromosome 7 amplification/chromosome 10 deletion is also one of the key genetic alterations that determine the DAG-G status, but the detection rate is relatively low in clinical practice. So, most of the patients enrolled in this study did not undergo relevant testing, and therefore this item was not identified and evaluated. In this regard, more patients with chromosome 7 amplification/chromosome 10 deletion information need to be included in future studies to further improve the completeness of the prediction model. Furthermore, the DAG-G prediction model established in this study requires further validation in multiple centers before it can be applied to the clinic.

## 13. Conclusions

The prediction model established based on preoperative MRI radiomics in this study can basically realize the prospective, non-invasive, and accurate diagnosis of DAG-G, which is of great significance to help optimize the treatment plan for such patients, including expanding the extent of surgery and actively administering radiotherapy, targeted therapy, or other treatments after surgery, so as to fundamentally maximize the prognosis of patients.

## Figures and Tables

**Table 1:** Brain MRI scanning parameters.

Sequence	Parameters
T1WI	TR = 2992 ms; TI = 869 ms; TE = 24 ms; Matrix = 320 × 320; FOV = 240 × 240 mm^2^; Thickness = 5 mm; Interval = 1.5 mm
T2WI	TR = 4599 ms; TE = 102 ms; Matrix = 320 × 224; FOV = 240 × 240 mm^2^; Thickness = 5 mm; Interval = 1.5 mm
T2-Flair	TR = 8000 ms; TI = 2100 ms; TE = 160 ms; Matrix = 256 × 256; FOV = 240 × 240 mm^2^; Thickness = 5 mm; Interval = 1.5 mm
DWI	TR = 4800 ms; TE = 74 ms; Matrix = 128 × 130; FOV = 240 × 240 mm^2^; Thickness = 8 mm; Interval = 0.94 mm
CE-T1WI	TR = 2992 ms; TI = 869 ms; TE = 24 ms; Matrix = 320 × 320; FOV = 240 × 240 mm^2^; Thickness = 5 mm; Interval = 1.5 mm

Abbreviations: TR = Repetition time; TI = Inversion time; TE = Echo time; FOV = Field of view.

**Table 2:** The baseline characteristics of enrolled patients.

Baseline Characteristics	Number of Astrocytoma Patients (Percentage)	Number of DAG-G Patients (Percentage)
Total	172	44
Gender		
Male	103 (60%)	30 (68%)
Female	69 (40%)	14 (32%)
Age (year)		
Mean ± standard deviation	47.97 ± 13.81	46.58 ± 12.55
Pathology grading		
WHO grade 2	105 (61%)	20 (45%)
WHO grade 3	67 (39%)	24 (55%)
IDH		
Mutant	85 (49%)	0 (0%)
Wild-type	87 (51%)	44 (100%)
TERT promoter		
Mutant	66 (38%)	31 (70%)
Non-mutant	106 (62%)	13 (30%)
EGFR		
Amplification	17 (10%)	14 (32%)
Non-amplification	155 (90%)	30 (68%)
Clinical symptom		
Epilepsy	88 (51%)	25 (57%)
Headache	23 (13%)	7 (16%)
Aphasia	22 (13%)	5 (11%)
Altered mental status	18 (10%)	3 (7%)
Others	21 (12%)	4 (9%)
Radiology manifestation		
-Hemisphere		
Left	79 (46%)	18 (41%)
Right	74 (43%)	23 (52%)
Both	19 (11%)	3 (7%)
-Location		
Frontal lobe	83 (48%)	22 (50%)
Temporal lobe	34 (20%)	10 (23%)
Parietal lobe	24 (14%)	5 (11%)
Insula	20 (12%)	4 (9%)
Others	11 (6%)	3 (7%)
-Contrast enhancement		
Non	92 (53%)	21 (48%)
Mild	35 (20%)	10 (23%)
Moderate	25 (15%)	7 (16%)
Ring-like	6 (3%)	2 (4%)
Heterogenous	14 (8%)	4 (9%)

Abbreviations: DAG-G = diffuse astrocytic glioma, IDH-wildtype, with molecular features of glioblastoma, WHO grade 4; IDH = Isocitrate dehydrogenase; TERT = Telomerase reverse transcriptase; EGFR = Epidermal growth factor receptor.

**Table 3:** Predictive performance of six classifiers for DAG-G.

Classifier	Average AUC	Average PRE	Average REC	Average F_1_
GNB	0.74 ± 0.07	0.68 ± 0.06	0.68 ± 0.08	0.67 ± 0.06
KNN	0.89 ± 0.04	0.83 ± 0.08	0.79 ± 0.12	0.79 ± 0.05
RF	0.96 ± 0.03	0.88 ± 0.06	0.87 ± 0.08	0.87 ± 0.05
AB	0.97 ± 0.02	0.91 ± 0.06	0.90 ± 0.08	0.89 ± 0.05
SVM	0.88 ± 0.05	0.85 ± 0.08	0.83 ± 0.10	0.83 ± 0.07
MLP	0.91 ± 0.03	0.87 ± 0.07	0.86 ± 0.08	0.86 ± 0.05

Abbreviations: DAG-G = diffuse astrocytic glioma, IDH-wildtype, with molecular features of glioblastoma, WHO grade 4; AUC = Area under curve; PRE = Precision; REC = Recall; F_1_ = F_1_-score; GNB = Gauss naive Bayes; KNN = K-nearest neighbors; RF = Random forest; AB = Adaptive boosting; SVM = Support vector machine; MLP = Multilayer perceptron.

## Data Availability

The data presented in this study are available on request from the corresponding author. The data are not publicly available to protect patient privacy.
